# New products

**DOI:** 10.1155/S1463924691000469

**Published:** 1991


Journal of Automatic Chemistry, Vol. 13, No. 6 (November–December 1991), pp. 289–294

### New products

### Laptop batteries

Gates Energy Products has a complete range of rechargeable nickel-cadmium batteries for portable computers. The Gates Valumax C system is a standard battery that provides a 25% capacity increase over previous standard products used in the laptop market. This translates into 3 to 4 h of runtime for users. The Valumax system features the proven Gates rechargeable nickel-cadmium technology in a standard C size with 2500 mAh capacity rating. Typical capacities obtained with this new cell in laptop applications have been at 2800 mAh. The VALUMAX line also includes other cell sizes of interest to designers of portable computers. The Valumax Cs (1600) mAh) and the Valumax 4/5 Cs (1200 mAh) are smaller and lighter in weight than the C cell, allowing designers to create a more compact and portable battery pack. The Valumax 650 mAh is a good choice for portable printers and other products with lower runtime requirements.

*Full information from Gates Energy Products, Dukes Court, Duke Street, Woking, Surrey GU21 5BH, UK. Tel.: 0483 740208.*

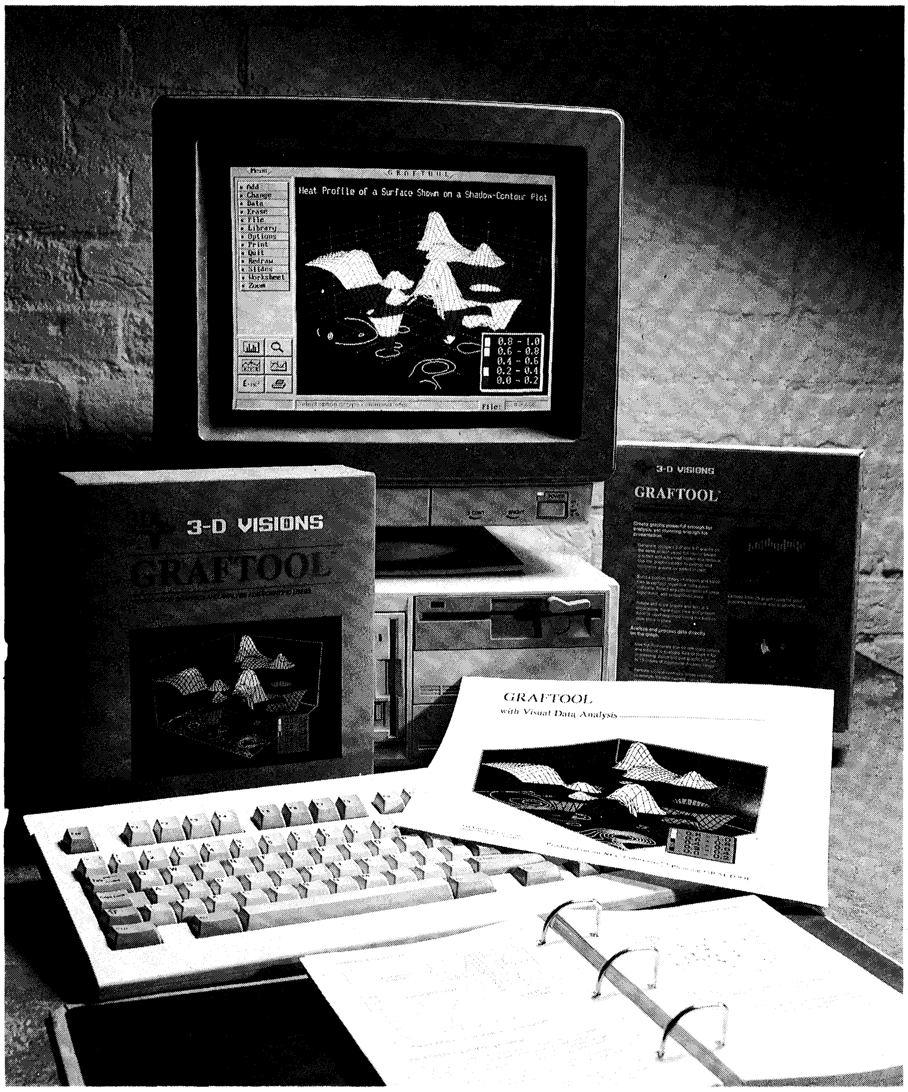

*Graftool.*

### Technical graphics program

Graftool is an intuitive package, with context-sensitve pop-up prompts, window-like dialogue boxes and simple mouse-button or menu selection. It offers full visual data analysis, so users can manipulate and process information and present it in a variety of ways. Unlike most standard graphics software, it has all the capability needed to handle the large and complex data sets generated in scientific and technical applications. It is a fully integrated 2D and 3D package, with a range of technical graphing functions from bar and pie charts to polar plots, scatter plots, vector plots and 3D surface plots. 3D to 2D data projection, contouring, surface gridding, cross-sectioning, point decomposition and colour mapping are all easily performed.

Graftool allows full user control of graph format, including the scaling and positioning of axes, text and annotation, arrows, frames and graphs. It provides zooming and panning at up to 15 orders of magnitude in dynamic range. Graphs can be rotated and resized, and colour-coded to aid assessment and analysis.

*Details from Adept Scientific Micro Systems Ltd, 6 Business Centre West, Avenue One, Letchworth, Hertfordshire SG6 2HB, UK.*

### Kjeldahl automation

The Kjeltec Auto Sampler System provides a high degree of automation whilst retaining flexibility. Up to 3 × 20 tubes can be loaded in the sampler for walk-away operation, while multitasking processors allow simultaneous analysis and further sample registration or result reporting. User definable programs allow the operator to select the degree of automation. For instance, sample weights can be entered directly from a balance, manually via the keyboard or run at constant weight.

The sampler allows up to 4 h of unattended operation, but also accommodates express samples.

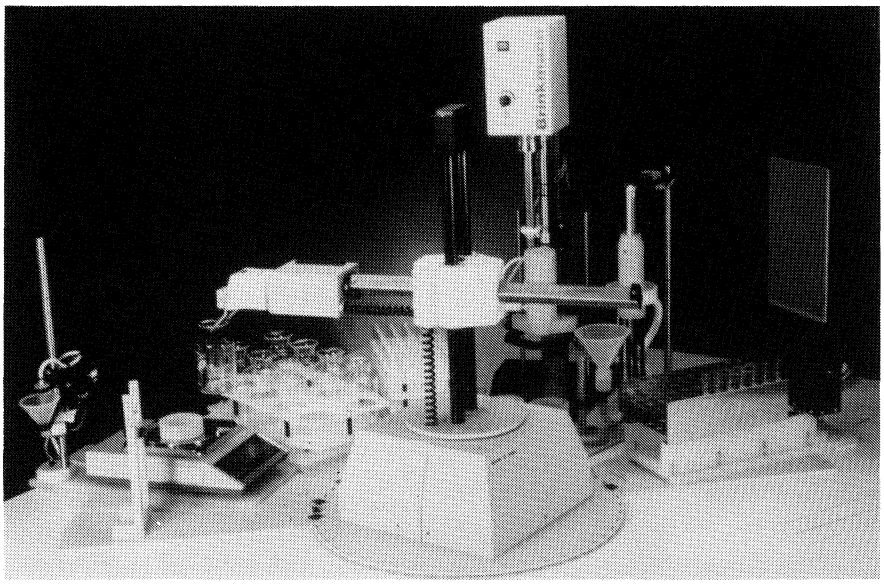

*Zymark has developed a dual workstation approach to automating tablet assay and content uniformity samples. The Tablet Processing Workstation, a general-purpose weighing and tablet dissolving workstation, automates the 'front end' of the tablet assay and content uniformity procedure. This is complemented by the BenchMate Workstation which wraps up the 'back end' of the procedure by completely automating sample filtration, dilution, and autosampling into a UV/Vis spectrophotometer or HPLC. The unique approach provides flexibility and the ability to handle multiple products and doses back-to-back within a single unattended run. Details from Sharon Correia, Zymark Corporation, Zymark Center, Hopkinton, Massachusetts 01748, USA.*

Remote PC control options and dump program assures flexible connection to LIMS-systems.

*Details from Tecator AB, Box 70, 263 21 Höganäs, Sweden. Tel.: 46 36 15 00.*

### Diluter/dispenser family

Hook and Tucker have introduced new liquid diluter/dispensers and can now offer models to cater for all liquid handling procedures. Firstly, there is the Compudil DR – a twin-syringe diluter/dispenser of similar specifications to the original Model D, but with the additional benefit of an RS232 interface for total computer handling applications, in addition to footswitch or handset operation at the laboratory bench.

With both the D and the DR version, there are 10 different syringes available to enable dispensing of any sample volume from 2–5000μl, and any diluent volume between 50 μl and 25 μl. Three interchangeable value blocks cover all options of syringe or tubing sizes.

Dispensing accuracy and precision are excellent. Actual volumes delivered are within 0·5% of desired volumes and typical performance gives CV better than 0·1%.

Volume selection is by a four-digit thumbwheel switch. Sample volumes can be selected in 1 μl steps, whilst diluent volumes are in 10 μl steps; and there are nine different operation speeds. Alternatively with the DR, an almost infinite range of operating procedures can be programmed via a PC.

The budget-priced Compudil DS is a single syringe version of the Compudil D with a similar technical specification and operating features. It offers dilution ratios of up to 1 : 250.

Lastly, and intended for routine laboratory situations which require accurate and precise liquid handling on-line, there is the new Compudil SR. This is a compact, single syringe instrument that is supplied with a choice of nine interchangeable syringes between 50 μl and 25 μl to give a wide choice of volumes and rations. It is driven by either a simple set of commands, via a RS232C serial link, or one of nine user-defined programs. Coupled with its inert fluid lines, these features make the unit amenable to remote operation in any environment.

The units are normally supplied ready for use on either 110 V AC 60 Hz or 220-240 V AC 50 Hz, but OEM versions can also be supplied without an outer case and/or power supply, and with motor driven rotary valve or solenoid operated valve.

*Details from Hook and Tucker Instruments, Vulcan Way, New Addington, Croydon CR0 9UG, UK. Tel.: 0689 843345.*

### Monochromator

The 0·25 m Czerny-Turner Monochromator is now available as a separate item for general applications. The optical design is based on a 0·25 m Czerny-Turner Monochromator with a 1200 l per mm grating blazed at 300 nm; giving a reciprocal dispersion of 3 nm/mm.

Wavelength selection is by means of stepper motor scanning system, which can be controlled either through a keypad with digital indication of wavelength, or through an IBM PC compatible computer, in which case the wavelength is shown on the computer screen. The monochromator housing is designed to accommodate a photomultiplier, thereby minimising stray light problems. The spectral range of the system is 186–930 nm and the aperture is $f/10$.

Six slit positions between 0·025 mm and 1·0 mm are provided. The slit mechanism is driven by a second stepping motor.

The Monochromator housing is provided with nitrogen purge facilities to maximize operation in the ultra-violet region.

*More information from ChemTech Analytical, 4 Railton Road, Wolseley Business Park, Kempston, Bedford MK42 7PN, UK. Tel.: 0234 843377.*

### DNA screening

Beckman's HDR (High Density Replicating) system performs multiple transfer of 96 clones, genomic DNA, or amplified samples from 96-well plates to blotting membranes.

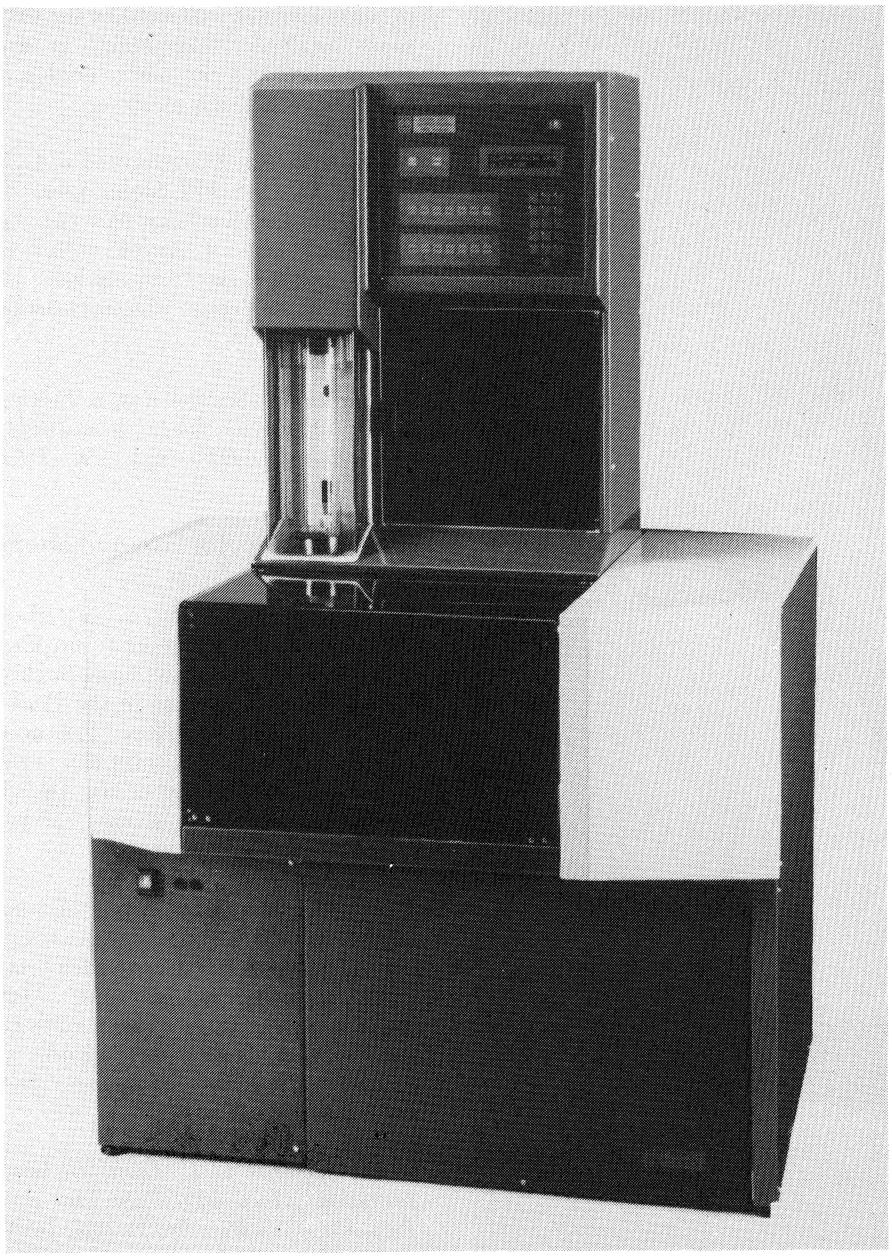

*The Kjeltec Auto Sampler System which handles up to 180 samples/day.*

Enhancing the applications of the Beckman Biomek 1000 to become a highly versatile DNA workstation, the high density grid saves both time and reagent cost when screening large volumes of DNA samples, and identifies the positive samples more efficiently than can be achieved by manual methods.

Utilizing stainless steel pins, the replicating tool produces high density blots for DNA screening. It precisely positions up to 1536 samples on a 7 × 11 cm membrane in approximately 30 min. Automated cleaning and sterilization of the tool between each transfer is included in this time cycle. As many as 96 samples per plate, up to 3456 samples using 36 plates, can be easily transferred.

The system is easily installed, and includes the HDR Tool with 0·015 in (0·381 mm) tip pins, bleach reservoir, water/ethanol reservoir module, fan unit and operating system. Pin kits are available with the 0·015 in tips or with 0·060 in (1·524 mm) tips in quantities of 100.

*Details from Beckman, Progress Road, Sands Industrial Estate, High Wycombe, Buckinghamshire, UK. Tel.: 0494 441181.*

### Oxidative stability of fats and oils

The Metrohm 679 Rancimat will oxidize in air, at elevated temperatures, samples of edible oils or fats. A conductivity measuring cell is used for the continuous detection of the volatile carboxylic acids liberated. The 679 then prints out the experimental curves continuously, on up to six samples at a time, using the inbuilt printer.

The Rancimat has a temperature range from 50 to 220 °C, allowing the determination of both sensitive and very stable oils.

In comparison with the traditional AOM test, the Rancimat method offers considerable savings in time. The AOM test also calls for periodic sampling and titrations which also often require sample treatment and is expensive.

Applications of the Rancimat are not only restricted to the rancidity of edible oils, but also to the processed and confectionery food industries, for example baby food, chocolate, ice cream etc.

*For further information contact V. A. Howe & Co. Ltd. Tel.: 0295 252 666.*

### Weighing and processing

Stevens Advanced Weighing Systems has restructured to concentrate on its business in the chemical/pharmaceutical and allied industries. Stevens Chemical Division has been created to allow a closer partnership between the Company and its customers because of the special needs of the chemical sector for systems capable of operation in aggressive environments and hazardous areas. Working within this new structure, the company believes it can help to reduce waste, increase efficiency, yield and quality at every stage of processing, from raw materials to packaged product.

*A brochure explaining the changes is available from Bryan Wall, Marketing Services Manager, Stevens Advanced Weighing Systems Ltd., Oak Industrial Park, Chelmsford Road, Dunmow, Essex CM6 1XN, UK.*

### Mass spectrometer

The Sciex (R) Model API III, from Perkin-Elmer, is a triple quadrupole

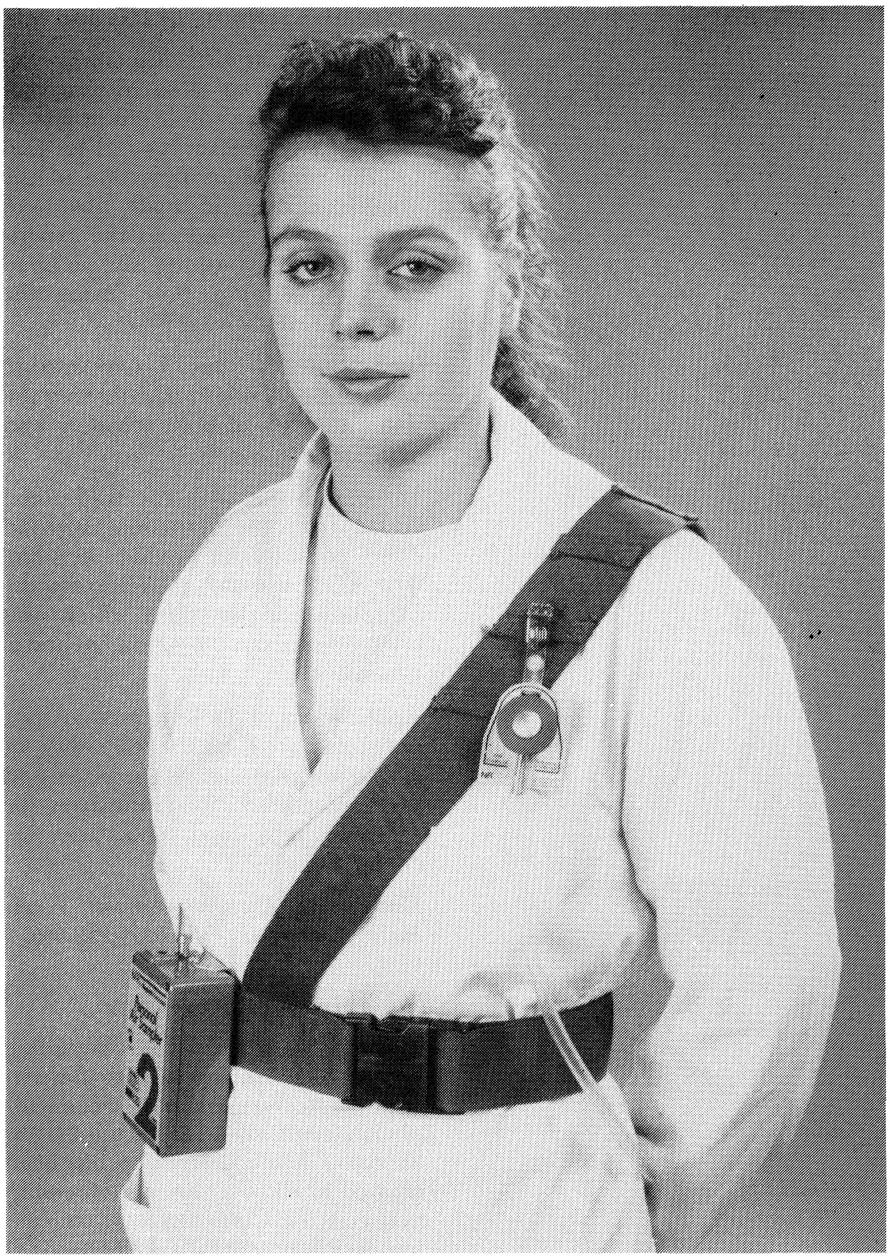

*Negretti Automation Ltd (manufacturer of the 2 l personal air-sampler above), based in Aylesbury, and OEH Scientific Ltd, based in Birmingham, have joined forces to provide a full air-sampling service. Companies having to undertake regular monitoring to comply with health and safety legislation, such as COSHH, will now be able to use the wide range of air samplers for dusts, fumes and gases supplied by Negretti Automation and have analytical work carried out on samples by OEH Scientific. More details from OEH Scientific, Aston Science Park, Love Lane, Aston Triangle, Birmingham B7 4BJ, UK. Tel.: 021 359 5361.*

mass spectometer linked to a liquid chromatograph for separation. Ions are created at atmospheric pressure, rather than inside the vacuum system, with little or no heating – a technique known as Atmospheric Pressure Ionization and applicable to polar, thermally labile molecules.

The ions produced are transferred to the mass spectrometer, which is maintained under high vacuum, by means of a specially-designed interface. This permits the ions to pass, while excluding the bulk of the sample and avoids the need to pump large volumes of solvent.

The technique is particularly appropriate to the analysis of pharmaceutical products, surfactants and fine chemicals.

The API III incorporates two mass analysers with a collision cell between them. This arrangement permits ions to be selected by the first mass analyser, fragmented in the collison cell and the pieces analysed by the second mass analyser.

The instrument is not limited to LC/MS or LC/MS/MS techniques, as liquid or dissolved samples can be introduced by a simple infusion pump, by flow injection analysis, or by capillary zone electrophoresis (CZE.)

*For further information contact Perkin-Elmer, Maxwell Road, Beaconsfield, Buckinghamshire HP9 1QA, UK. Tel.: 0494 676161.*

### Rotor carrier for round-bottom tubes

Beckman Bulletin DS-787 describes a new 4 × 50 ml general-purpose carrier for the JS-7.5 swinging bucket centrifuge rotor for use in the Beckman J2-21 and J6 Series. The new carrier requires no adapters and increases productivity of the JS-7.5 by accommodating sixteen 50-ml round-bottom sample tubes.

The bulletin includes a photograph of the rotor with the new carriers installed, providing users with a total rotor capacity of 800 ml. Also provided are a table of performance specifications and part numbers, simplifying product evaluation and order placement.

*Reader enquiries to L. Croft, Beckman Instruments, Inc., 1050 Page Mill Rd., Palo Alto, California 94303-0803, USA.*

### Regulation

Two new data sheets from Philips Analytical highlight instrumentation designed for Good Laboratory Practice (GLP) compliance, together with the broader issue of quality and accreditation standards.

'The PU8730 Series UV/visible spectrometer in the GLP environment' identifies features of the PU8730 which will help laboratories – especially in pharmaceutical and allied industries – to comply with GLP. Regulatory procedures are playing an increasingly important role in the analytical laboratory, with GLP ensuring that laboratory work is planned, performed, monitored, recorded and reported so that the

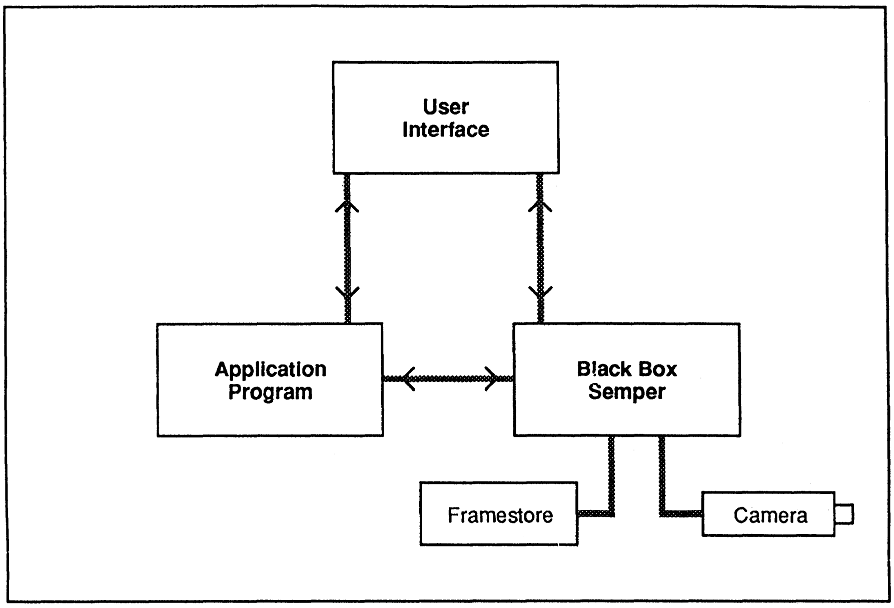

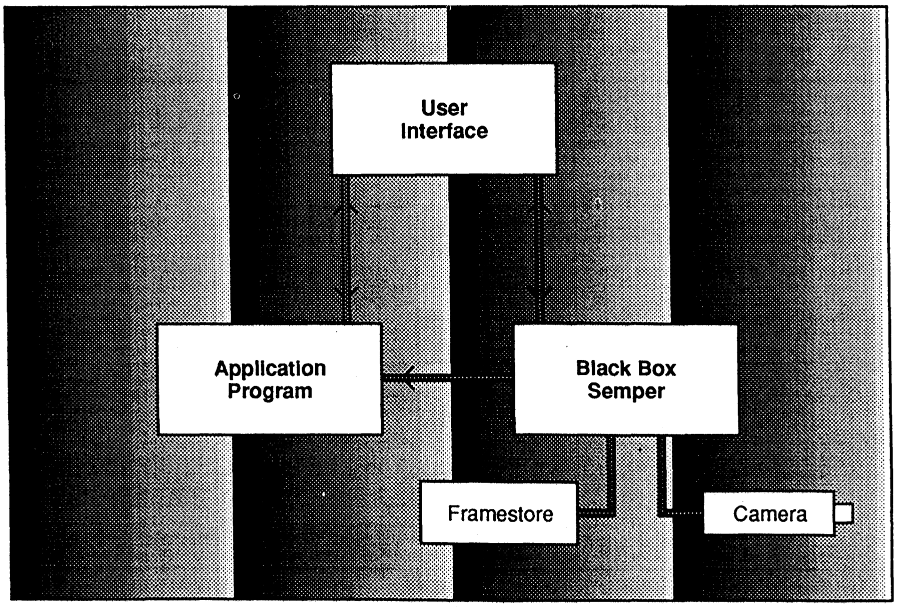

*The Black Box Semper is a self-contained image processing system with simple communication handles to the outside world and is an innovation for the image processing OEM. Targeted at third party software developers, Black Box Semper provides a sophisticated image processing server and multi-tasking system for a client application. It offers a complete development environment in which the image processing functions are independent of the final product, allowing the developer to focus on his own application.*

*As a first release, Black Box Semper is available on a PC running Windows 3.0 and will ultimately be hardware independent, allowing freedom of choice over host computer, display device, framestore and camera. This flexibility extends to the unlimited choice of graphical user interface which is used with the developers' software, so that Black Box Semper remains hidden from the user. An example graphical interface is provided by Synoptics on Windows 3.0. The schematic illustration is of a Black Box Semper; the second diagram is identical to the first, but is superimposed against a text pattern.*

*At the application program level, this product provides an ideal programming and algorithm development environment. The communication protocol is established by the black box so that the developer can access its image processing functionality using simple 'C' subroutines. Programs written in Semper can be accessed through control messages sent as ASCII strings. Black Box Semper runs the program and produces a result which is then sent back to the host program thus eliminating the need to write volumes of code in a low-level language.*

*Details from Synoptics Ltd, 271 Cambridge Science Park, Milton Road, Cambridge, CB4 4WE, UK. Tel.: 0223 423223.*

integrity and validity of results is assured.

'Quality and accreditation standards: a comparative review' assesses GLP against ISO 9000 (UK designation BS 5750) and NAMAS (National Measurement Accreditation Service) for quality systems and analytical data.

Analytical instrument manufacturers and testing facilites are increasingly aware of the importance of recognized European accreditation in preparation for the Single Market – Philips Analytical's registration to ISO 9001 (BS 5750 Part 1) parallels this process, encoding company quality which is traceable to an internationally recognised standard.

*More information from Paul Carter, Philips Analytical, York Street, Cambridge CB1 2PX, UK. Tel.: 0223 358866.*

### Particle size

INSITEC'S EPCS particle analyser fits directly into the process stream via flange connection (pipes 6 in or larger). This rugged, real-time, instrument provides 100% representational data for total quality control and product monitoring. Remote operation increases safety and reduces risk of product contamination. This system measures size (general range 1 to 1000 microns) and concentration (up to 1000 gm/$m^3$) of irregular or spherically shaped powders. It can operate at velocities from zero to supersonic. If particles are within this size range and are pneumatically conveyed they can now be measured *in situ*. A probe version of this system has been designed for use as a source monitor for stack emissions. Software records data at user established intervals for quality verification and analysis.

*Details from Janice Persons, INSITEC, Inc., 2110 Omega Road, Suite D, San Ramon, California 94583, USA. Tel.: 415 837 1330.*

### Chromatography manager

A colour brochure describes Axxiom Chromatography's PYRAMID Chromatography Manager, a user-configurable data acquisition and

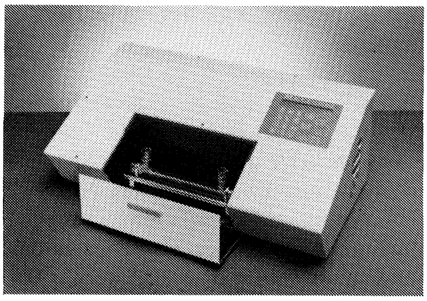

*Optical Activity Ltd's new programmable Polarimeter: the PolAAr 21. The PolAAr 21 offers built-in programming, six measurement scales, wide operating range and interfaces to automatic data collection systems. These features make it suitable for either analytical or continuous process monitoring work. Details from Optical Activity Ltd, Bury Road Industiral Estate, Ramsey, Cambridgeshire PE17 1NA. Tel.: 0487 813913.*

analysis system. The brochure lists PYRAMID's features, including graphic programming and file editing, full configurability of all displays and reports, and direct control of HPLC and GC instruments from most manufacturers. It also lists computer requirements for installing and running this PC-based, Windows 3.0 system. Both versions of PYRAMID, for managing either one or two chromatographs, are reviewed. A brief section concerning PYRAMID's GLP Mode operation explains features which provide total, automatic GLP/GMP compliance.

*Copies from Axxiom Chromatography, Inc. 11988 Challenger Court, Moorpark, California 93021-7122, USA. Tel.: 805 523 8888.*

### Flow-injection analyser

Burkard Scientific have recently launched a flexible system flow-injection analyser. The FIA-FLO is an automatic single or dual valve system which provides total flexibility to the user. It is recommended for use in such areas as monitoring sea and river water pollution, including tests for aluminium, chemical or process plant monitoring; food processing and manufacture; pharmaceutical quality control; and research.

The FIA-FLO includes a CF1-X or CFX-2 and CF1-UV ultra-violet detector. The sample changer is available with a 40-place capacity or serpentine with up to 300 sample cups. An X-Y sampler option is also offered.

A data analyser is also available which includes a 40 MByte hard disk and high resolution colour monitor, and single or dual channel recorder. Features available include auto start-up and closedown. Results can be presented in a variety of modes and can be fed to spreadsheet or database management software.

*More information from Mr B. G. Wili, Burkard Scientific (Sales) Ltd, P.O. Box 55, Trading Estate, Eskdale Road, Uxbridge, Middlesex UB8 2RT, UK. Tel.: 0895 300056*

### Dyeing

The Metrohm 665 Dosimat checks dye deliveries against a standard for colour and strength, and the preparation of formulations for colours to be produced by the bulk, dyeing process must be performed with a high level of accuracy and reproducibility.

The 665 Dosimat offers a high degree of accuracy, and a high speed of operation at a relatively low cost; in addition, it requires little space and can be placed in a laminar flow cabinet (COSHH requirements). The exchange unit system ensures easy change of solvent/water supply.

*For further information contact V. A. Howe & Co. Ltd, Beaumont Close, Banbury, Oxfordshire OX16 7RG, UK. Tel.: 0295 252666.*

